# Distribution of Mercury in Rainbow Trout Tissues at Embryo-Larval and Juvenile Stages

**DOI:** 10.1100/2012/652496

**Published:** 2012-05-02

**Authors:** Renáta Kenšová, Kamila Kružíková, Jan Havránek, Danka Haruštiaková, Zdeňka Svobodová

**Affiliations:** ^1^Department of Veterinary Public Health and Toxicology, Faculty of Veterinary Hygiene and Ecology, University of Veterinary and Pharmaceutical Sciences Brno, Palackého 1/3, 612 42 Brno, Czech Republic; ^2^Research Centre for Toxic Compounds in the Environment, Masaryk University, Kamenice 126/3, 625 00 Brno, Czech Republic

## Abstract

The aims of the study were to determine total mercury concentrations in “rainbow trout *Oncorhynchus mykiss* (Walbaum)” at their embryo-larval and juvenile stages and to assess mercury concentration dynamics in individual tissues. Samples of rainbow trout were collected at two-month intervals over a period of 18 months (one stock production cycle) at the Velká Losenice trout farm. Feedstuff samples were collected at the same time and analyzed for mercury concentrations. Tissue mercury concentrations were determined in muscle, liver, and kidneys. Analyses were performed using the AMA 254 atomic absorption spectrophotometer. The lowest mercury concentration was found in 14-day-old embryos (hard roe), and the highest concentrations in muscle tissue, liver, and kidneys at the end of monitoring, that is, in rainbow trout aged 18 months. The amount of mercury in feedstuffs showed an increasing trend and ranged between 0.0126 and 0.0859 mgkg^−1^. A significant effect (*P* < 0.001) of mercury intake on mercury concentrations in muscle tissue, liver, and kidneys was demonstrated. Muscle mercury concentrations in 18-month-old market-ready rainbow trout of 0.128 ± 0.048 mgkg^−1^ met the criteria for fish meat hygiene.

## 1. Introduction

Heavy metals are not as a rule primarily lethal for aquatic animals. However, their long-term negative effects cause developmental defects in fish, reproductive defects, and immunosuppression, and they may also cause ecological instability of aquatic ecosystems [1]. Growing heavy metal concentrations in the environment and subsequently in foods have become a major hygienic issue. In recent years, food safety has become a priority in EU member states. For a long time, mercury has been among the most closely monitored elements in fish because of its high toxicity and ability to accumulate. Mercury is also used as an indicator of environmental contamination with industrial waste [2–4]. It is a global contaminant distributed in the environment including organisms. The most toxic form of mercury for the human organism is organic methymercury [1]. As far as fish as animal for food production are concerned, the main attention is focussed on economically important species (carp, rainbow trout) [3].

The aims of the present study were to determine total mercury concentrations in rainbow trout at their embryo-larval and juvenile stages, to assess mercury concentration dynamics in individual tissues, and to find out whether mercury levels in fish correlated with mercury levels in their feedstuffs. 

## 2. Material and Methods

### 2.1. Fish Sampling

Between 2008 and 2009, samples of rainbow trout *(Oncorhynchus mykiss)* ranging from hard row (A) to harvest-size fish (H) were collected at the Velká Losenice trout farm at regular intervals over the 18-month production cycle. Six groups had 10 fish each, while groups H and A consisted of 5 fish and 4 mixed embryo groups, respectively. Characteristics of individual groups, date of sample collection, age, number of fish, total body length, and weight of fish are summarized in Table 1. We set the interval between sample collections at two months. The extended interval between groups E and F sample collections was due to the winter resting period.

**Figure 1:**
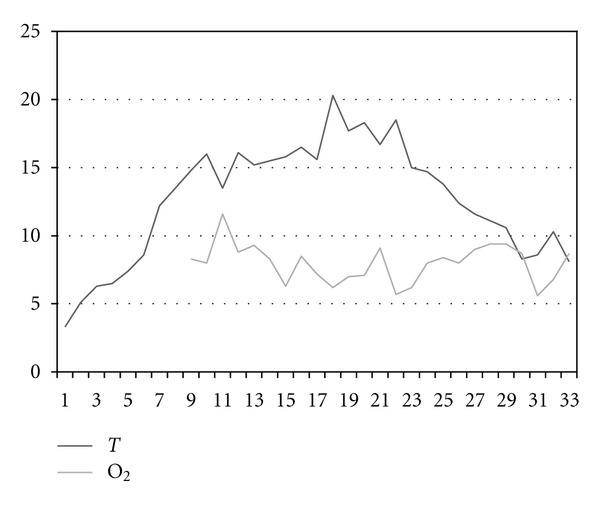
Water temperature (°C) and oxygen concentration (mg·l^−1^) in 2008 (weeks 1–33).

**Figure 2:**
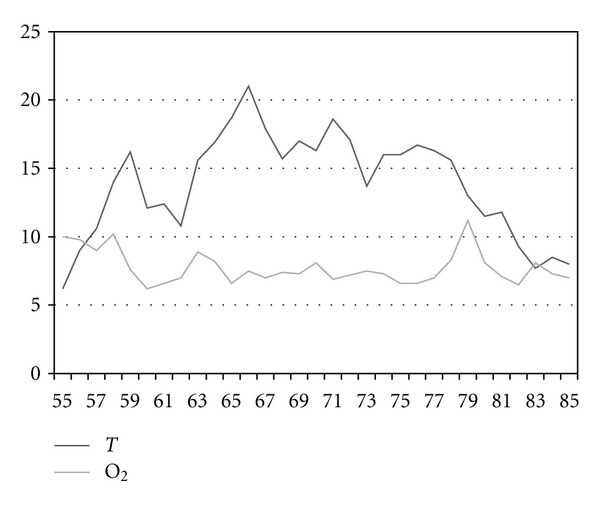
Water temperature (°C) and oxygen concentration (mg·l^−1^) in 2009 (weeks 55–85).

The trout farm in Velká Losenice is part of the fish farming company Rybářství Velké Meziříčí a. s. in the Žd'ár nad Sázavou district. The farm is situated in the upper reaches of the River Sázava in sparsely populated and forested area, free of any anthropogenic water pollution. Water temperature and oxygen saturation are showen in Figures 1 and 2.

Health status of fish was checked immediately after they were caught. The fish were then sacrificed, measured, and weighed, and samples of individual tissues (muscle, liver, kidneys and, in Group H, also gonads) were collected. Because of low weight and therefore small size of internal organs, whole fish from groups B and C were cold-stored and whole-body homogenates were used for the analysis. The specimens collected were airtight sealed in microtene bags, frozen, and kept in a deep freezer at –86°C until the analysis was performed.

### 2.2. Feedstuff Sampling

Samples of feeds fed to the fish were collected at the same time as fish samples (Table 2). Feedstuff number 1 was fed to fry and to the smallest fish categories, that is, in the period from April 2008 to August 2008. Over the next period of about a month, feedstuff number 2 was gradually introduced to the feed ration, and it continued to be fed until the beginning of the winter resting period, that is, until November 2008. In the period of transition from one feedstuff to another, that is, for 3-4 weeks, a mix of the two feed types was used. From their resting period from December 2008 to February 2009, the fish were either made to fast or were fed only minimum amounts of Feedstuff number 2. 

In the second year of the production cycle (2009), the fish were fed Feedstuff number 3 throughout the entire period starting in March and ending in late summer 2009 when they were shipped. The quantities of feedstuffs fed to the fish calculated according to the weight of fish stock, water temperature, O_2_ concentrations, and the season of the year were in the range of 0.25–2% fish stock weight (Table 3).

### 2.3. Determination of Total Mercury Concentrations

The single-purpose atomic absorption spectrophotometer AMA 254 (Altec s.r.o., CZ) with the detection limit of 0.01 ng Hg was used to determine total mercury concentrations in individual fish tissues and in feedstuffs. The instrument is intended for direct mercury determinations in solid and liquid samples without chemical pretreatment of samples (mineralization, etc.).

Total mercury concentrations in fish tissues are given in mgkg^−1^ fresh tissue and total mercury concentrations in feedstuffs in mgkg^−1^ of feedstuffs analyzed (moisture of feedstuff was 9%).

### 2.4. Determination of Mercury Intake

Average feedstuff mercury concentrations were 0.0126 mgkg^−1^ (sample 1), 0.0401 mgkg^−1^ (sample 2), and 0.0859 mgkg^−1^ (sample 3). The values are means of three parallel measurements. The maximum permitted limit for mercury in complete feeds is 0.1 mgkg^−1^ (Decree 356/2008 Sb) [5]. This act includes many limits for contaminating compound in feed. In our study, mercury levels in the feedstuff used did not exceed feed hygiene limits. The mercury in feedstuff was naturally present, and the main of feedstuff is fish powder. That is why mercury can come just only from this fish powder and no mercury was added during the experiment.

**Figure 3:**
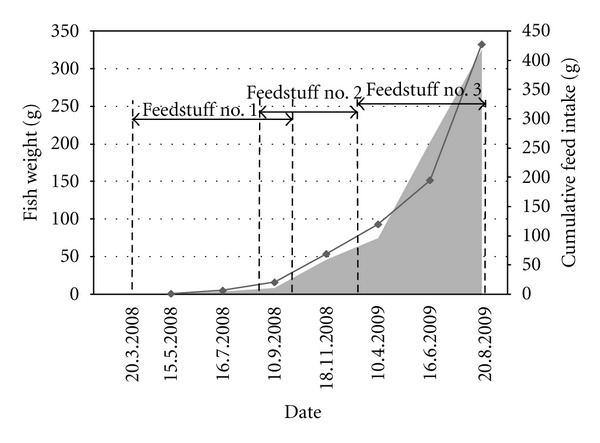
Fish growth curve (solid line), cumulative feed intake (grey area), and feeding period of different feedstuff types.

Average values of fish weight within age groups were used to construct growth curve providing the fish weight for every day of the monitoring period (Figure 3). The information from growth curve and feeding rates (percent of body weight to be fed each day, Table 3) was used to calculate feed intake. Finally, amount of mercury intake was calculated as the feed intake multiplied by mercury concentrations in feedstuffs (Table 3). Cumulative amount of mercury intake takes into account information on fish weight and fish age as well as on mercury concentration in feedstuff.

### 2.5. Validation and Statistical Evaluation

The accuracy of the method used for total mercury determination was validated using BCR number 278 Muscle Tissue standard reference material.

A total of 69 fish from eight age groups (A–H) were analyzed for mercury concentrations (Table 1). Mercury concentration in muscle, liver and kidney was measured in 45 fish (age groups D–H). One another mercury parameter was derived as the ratio of mercury concentration in liver and in muscle.

All the variables were tested for normal distribution within age groups by means of the Shapiro-Wilk test. Since all the variables fit the normal distribution, data were analyzed using parametric methods. Mercury concentrations in muscle, liver, and kidney within each age group were compared using a set of *t*-tests for dependent samples. Because *t*-tests were used three times to test one hypothesis, the significance level was adjusted to *α* = 0.0167 (Bonferroni's correction).

Linear regression was performed to control for the effect of cumulative amount of mercury intake on mercury concentration in muscle, liver, kidney, and ratio of mercury concentration in liver and in muscle. Simple linear regression analysis was more appropriate than multiple regression analysis including all the independent variables (fish age, fish weight, cumulative amount of mercury intake) because these variables were highly correlated (fish age and fish weight: *r* = 0.846, *P* < 0.001; fish age and cumulative amount of mercury intake: 0.827, *P* < 0.001, fish weight and cumulative amount of mercury intake: 0.933, *P* < 0.001). In such cases with redundant information in the regression model, the model suffers under less exact estimates and very complicated interpretation. Moreover, the cumulative amount of mercury intake is a complex variable involving information on fish age, fish weight, and mercury concentration in feedstuff.

Data analyses were performed using Statistica software [6].

## 3. Results

**Figure 4:**
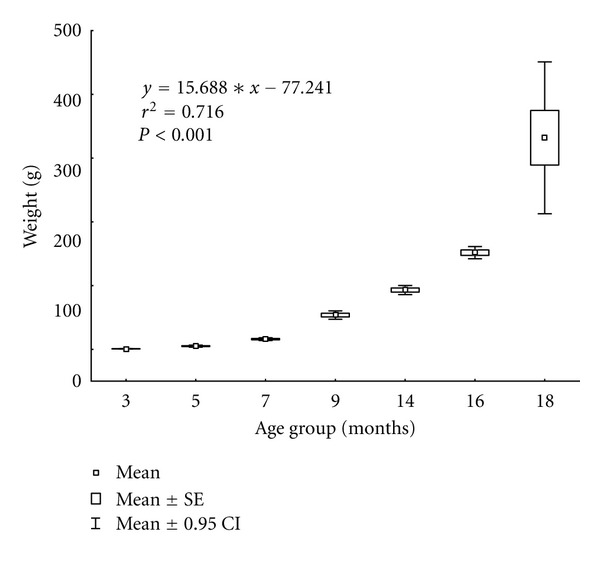
Fish weight during the monitoring period.

Figure 4 showing weights of fish in the stock analyzed throughout the production cycle demonstrates the continuous growth of the fish, slightly slowed down in the winter period, and accelerated at the end of the cycle when feed intake levels and weight gains are the highest. Differences in the weight of fish from different age groups were statistically significant (*P* < 0.001). Similarly, differences in total body length between different age groups were also statistically significant (*P* < 0.001). By the end of the 18-month period of monitoring, the fish had reached market size.

Mercury concentrations in tissues investigated are summarized in Table 4. Because of low age and size of fish in groups A–C, it was impossible to determine mercury concentrations in their individual tissues so embryo or whole-body homogenates were used.

The lowest mercury concentrations in all tissues were in group D (age 7 months) and the highest in group H (age 18 months). The same holds true for the liver/muscle mercury ratio (Table 4).

**Figure 5:**
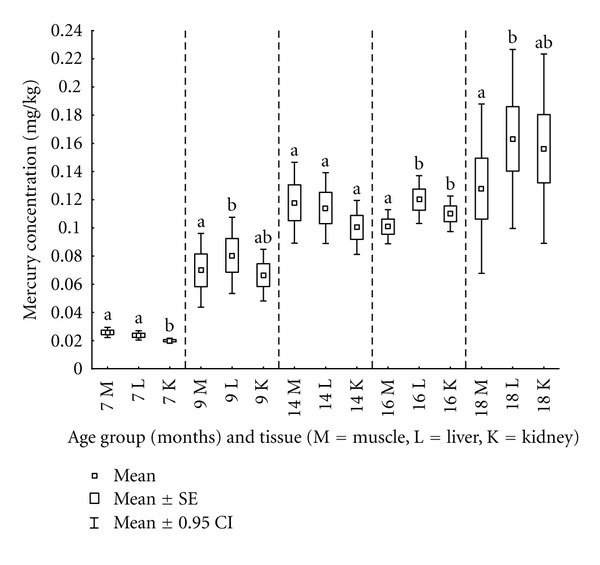
Mercury concentrations in muscle, liver, and kidney in individual age groups.

Average mercury concentrations in muscle, liver, and kidney of fish aged 7 months were 0.026 mgkg^−1^, 0.024 mgkg^−1^, and 0.020 mgkg^−1^, respectively (Table 4, Figure 5). Kidney mercury concentrations differed significantly from muscle and liver mercury concentrations (*t*-test: muscle—kidney: *t* = 6.474, df = 9, *P* < 0.001; liver—kidney: *t* = 4.548, df = 9, *P* = 0.001; Figure 5).

Muscle and liver mercury concentrations in fish aged 9 months differed significantly (*t*-test: muscle—liver: *t* = −5.596, df = 9, *P* < 0.001; Figure 5). The highest concentration was found in liver (0.081 mgkg^−1^), muscle concentrations were lower (0.070 mgkg^−1^), and kidney concentrations were the lowest (0.067 mgkg^−1^; Table 4).

Muscle, liver, and kidney mercury concentrations in fish aged 14 months were 0.118 mgkg^−1^, 0.114 mgkg^−1^, and 0.100 mgkg^−1^, respectively (Table 4, Figure 5). These differences were not statistically significant.

The muscle mercury concentration (0.101 mgkg^−1^) was significantly lower than liver and kidney mercury concentrations (0.120 mgkg^−1^ and 0.110 mgkg^−1^, resp.) in fish aged 16 months (*t*-test: muscle—liver: *t* = −7. 169, df = 9, *P* < 0.001; muscle—kidney: *t* = −2.996, df = 9, *P* = 0.015; Figure 5).

In fish aged 18 months, the liver mercury concentration (0.163 mgkg^−1^) was significantly higher than the muscle mercury concentration (0.128 mgkg^−1^; *t*-test: muscle—liver: *t* = −5.826, df = 4, *P* = 0.004; Table 4, Figure 5). Total mercury levels in gonads (0.029 mgkg^−1^) were determined only in group H, the last age group monitored, where it was already possible to collect samples.

Mercury levels in the A sample—embryos (0.004 mgkg^−1^) were markedly lower than in other samples. All the samples confirm that mercury levels increase in the course of the fish rearing cycle.

Liver/muscle ratios in individual groups are summarized in Table 4. Ratios exceeding 1 indicate that mercury from feedstuffs is preferentially deposited in the liver and only then transported to the muscle tissue. Such values were ascertained in periods of the most intensive feed intake, and involved groups E (9 months), G (16 months) and H (18 months). In groups D (7 months) and F (14 months), however, the ratios were almost equal to 1 or were below 1. In these groups, there was a reduction in feed intake in the previous period. Mercury had already been redistributed to the muscle tissue, and further reception of mercury and its deposition in the liver was reduced. They were groups of fish after the winter resting period (month 14) or in summer months (month 7) when water temperatures exceeded 20°C, which caused feed intake reduction.

Mercury concentration in muscle and liver as well as in kidney was significantly affected by cumulative amount of mercury intake (*P* < 0.001, *r* ^2^ varied from 0.253 to 0.533, Table 5). Mercury concentration in fish tissues increased with increasing mercury intake. The same holds true for liver/muscle mercury concentration ratio (*P* = 0.001, *r* ^2^ = 0.222; Table 5).

## 4. Discussion

In a large majority of published studies on heavy metal concentrations in fish, concentrations of the metals were monitored only in individual fish or groups of fish captured for the purpose of monitoring environmental contamination or food quality [4, 7–12]. The present paper reports results of investigations into mercury concentrations of rainbow trout reared for commercial purposes on a trout farm. The monitoring continued for the entire rearing period during which 8 groups of samples were collected at two-month intervals (one of the intervals was 5 months when one sampling during the winter resting period was skipped). Three different types of feedstuffs of increasing mercury levels were used over the 18-month rearing period. Because the rainbow trout were farmed in an area free of any significant anthropogenic contamination, the main source of growing mercury tissue concentrations was the feedstuffs. The last group of fish evaluated were harvest-size fish intended for consumption, and attention is therefore given here also to the food hygiene aspects. At present, hygienic limits are determined by the highest acceptable levels of contaminants in foodstuffs in the Commission Regulation 1881/2006/EC as amended [13]. The above regulation set the maximum mercury level for fishery products and fish muscle meat at 0.5 mgkg^−1^ (also applies to rainbow trout) and 1.0 mgkg^−1^ for selected fish species (mainly predatory fish species). Total mercury concentrations found in our study never exceeded or even approached that limit. The highest total mercury concentration in muscle was found in group H at 18 months (0.128 ± 0.048 mgkg^−1^).

Mercury concentrations in rainbow trout tissues were studied by Ciardullo et al. [14] and Arribére et al. [15]. Ciardullo et al. [14] determined mercury concentrations in tissues of rainbow trout aged 10–14 months. They found the highest mercury levels in the kidneys, followed by gills, muscle, liver, and skin. Muscle mercury concentrations were independent of fish weight. The lowest and the highest kidney mercury concentrations were ascertained in the kidneys at 14 and 40 months, respectively. Similar concentration was ascertained in the liver, except that the lowest mercury concentration there was found at 16 months. The highest mercury concentrations in the oldest fish group (40 months) were in the kidneys and liver and the lowest in the skin. Our study, on the contrary, confirmed a correlation between tissue mercury concentrations and fish weight and age when the highest mercury concentrations in all tissues analyzed were ascertained in the oldest age group (18 months). 

Arribére et al. [15] monitored Hg concentrations in the liver and muscle of fish captured in summer and winter in the lakes of two national parks in Argentina. One of the sites monitored was the rainbow trout farm of the university. On that farm, trout were captured in spring at the age of 2 years. The liver/muscle mercury ratio found there was 1.140, which suggests preferential deposition of mercury in liver. We found a similar situation in our study in fish from group G (16 months) from the same season of the year. Their liver/muscle mercury ratio was 1.185, which suggests preferential deposition of mercury in liver and its later transport to muscle. Both fish groups were in the period of elevated feed intake.

A significant effect of cumulative amount of mercury intake on mercury concentrations in fish tissues was demonstrated. Also demonstrated was linear growth in mercury concentrations in muscle, liver, and kidneys in the course of embryo-larval and juvenile stages. Because the study was conducted on a farm free of any significant source of anthropogenic contamination, the main source of growing mercury tissue concentrations was the feedstuffs. Mercury concentrations found in harvest-size trout fully meet the existing hygiene limit (0.5 mgkg^−1^).

## Figures and Tables

**Table 1:** Characteristics of fish groups analyzed.

Group	Sampling date	Age (months)	*n*	Total body length (mm)	Weight (g)
A	20. 3. 2008	0.5	4*	—	—
B	15. 5. 2008	3	10	40 ± 8	0.9 ± 0.5
C	16. 7. 2008	5	10	72 ± 8	5.1 ± 2.2
D	10. 9. 2008	7	10	106 ± 7	16.0 ± 3.1
E	18. 11. 2008	9	10	168 ± 13	53.7 ± 9.1
F	10. 4. 2009	14	10	205 ± 13	93.0 ± 10.0
G	16. 6. 2009	16	10	235 ± 11	151.6 ± 13.5
H	20. 8. 2009	18	5	301 ± 26	332.0 ± 96.0

*4 mixed embryo samples were collected.

**Table 2:** Samples of feedstuffs used.

Sample number	Sampling dates	Manufactured by	Trade name	Granule size	Batch
1	15. 5. 2008	Biomar A/S	Aquastart 2	2 mm	73971
2	16. 7. 2008	Biomar A/S	Aqualife 17	3 mm	69152
3	10. 4. 2009	Biomar A/S	Aqualife 17	4.5 mm	74080

**Table 3:** Feeding rates (percent of body weight per day) and feedstuff types during the sampling period.

Date	Feeding rates (%BW/day)	Feedstuff	Feedstuff mercury concentration (mgkg^−1^)
20.3.–15.5.2008	2	Sample number 1	0.0126
16.5.–16.7.2008	2	Sample number 1	0.0126
17.7.–10.9.2008	1	Sample number 1, sample number 2*	0.0126–0.0401*
11.9.–18.11.2008	2	Sample number 1, sample number 2*	0.0126–0.0401*
19.11.2008–10.4.2009	0.5 (0.25)^+^	Sample number 2, sample number 3°	0.0401–0.0859°
11.4.–16.6.2009	2	Sample number 3	0.0859
17.6.–20.8.2009	1	Sample number 3	0.0859

*Gradual transition of feedstuff sample 1 to feedstuff sample 2 in September 2008.

^+^Fish fed only 0.25% BW/day of feedstuff number 2 in resting period (December 2008–February 2009).

°Feedstuff number 2 till February 2009, feedstuff number 3 from March 2009.

**Table 4:** Total mercury concentrations found in individual tissues investigated.

	Mercury concentration (mgkg^−1^) in individual tissues (*x* ± SD)					
Group (month)	Embryos	Whole-body homogenate	Muscle	Liver	Kidney	Liver/muscle ratio
A 0.5	0.004 ± 0.003	—	—	—	—	—
B 3	—	0.039 ± 0.019	—	—	—	—
C 5	—	0.043 ± 0.012	—	—	—	—
D 7	—	—	0.026 ± 0.005	0.024 ± 0.005	0.020 ± 0.002	0.930 ± 0.134
E 9	—	—	0.070 ± 0.037	0.081 ± 0.038	0.067 ± 0.026	1.199 ± 0.133
F 14	—	—	0.118 ± 0.040	0.114 ± 0.035	0.100 ± 0.027	1.010 ± 0.244
G 16	—	—	0.101 ± 0.017	0.120 ± 0.024	0.110 ± 0.018	1.185 ± 0.068
H 18	—	—	0.128 ± 0.048	0.163 ± 0.051	0.156 ± 0.054	1.305 ± 0.145

**Table 5:** Effect of cumulative amount of mercury intake (*x*) on mercury concentration in muscle, liver, kidney, and liver and muscle mercury concentration ratio. (*N* = 45).

	Regression equation	*r* ^2^	*P*
Hg in muscle	0.064 + 0.002*x	0.253	<0.001
Hg in liver	0.063 + 0.003*x	0.439	<0.001
Hg in kidney	0.053 + 0.003*x	0.533	<0.001
Hg in liver/Hg in muscle	1.026 + 0.009*x	0.222	0.001

## References

[1] Eisler R (2006). *Mercury Hazards to Living Organisms*.

[2] Svobodová Z, Máchová J, Vykusová B, Piačka V (1996). Metals in ecosystems of surface water. *Methods RIFCH Vodňany*.

[3] Svobodová Z, Hejtmánek M, Studnicka M, Randák T (2004). Mercury content in muscle of economically important fish species in the Czech Republic—review. *Veterinářství *.

[4] Houserová P, Kubáň V, Spurný P, Habarta P (2006). Determination of total mercury and mercury species in fish and aquatic ecosystems of Moravian rivers. *Vet Med-Czech*.

[6] StatSoft (2007). STATISTICA (data analysis software system), version 8.0.. http://www.statsoft.com.

[7] Kružíková K, Randák T, Kenšová R, Kroupová H, Leontovyčová D, Svobodová Z (2008). Mercury and methylmercury concentrations in muscle tissue of fish caught in major rivers of the Czech Republic. *Acta Veterinaria Brno*.

[8] Havelková M, Dušek L, Némethová D, Poleszczuk G, Svobodová Z (2008). Comparison of mercury distribution between liver and muscle—a biomonitoring of fish from lightly and heavily contaminated localities. *Sensors*.

[9] Marrugo-Negrete J, Verbel JO, Ceballos EL, Benitez LN (2008). Total mercury and methylmercury concentration in fish from the Mojana region of Columbia. *Environ Geochem Health*.

[10] Vítek T, Spurný P, Mareš J, Ziková A (2007). Heavy metal contamination of the Loučka river water ecosystem. *Acta Veterinaria Brno*.

[11] Maršálek P, Svobodová Z, Randák T (2006). Total mercury and methylmercury contamination in fish from various sites along the Elbe river. *Acta Veterinaria Brno*.

[12] Staniskiene B, Matusevicius P, Budreckiene R, Skibniewska KA (2006). Distribution of heavy metals in tissues of freshwater fish in Lithuania. *Polish Journal of Environmental Studies*.

[14] Ciardullo S, Aureli F, Coni E (2008). Bioaccumulation potential of dietary arsenic, cadmium, lead, mercury, and selenium in organs and tissues of rainbow trout (*Oncorhyncus mykiss*) as a function of fish growth. *Journal of Agricultural and Food Chemistry*.

[15] Arribére MA, Guevara SR, Bubach DF, Arcagni M, Vigliano PH (2008). Selenium and mercury in native and introduced fish species of Patagonian Lakes, Argentina. *Biological Trace Element Research*.

